# ChemOS: An orchestration software to democratize autonomous discovery

**DOI:** 10.1371/journal.pone.0229862

**Published:** 2020-04-16

**Authors:** Loïc M. Roch, Florian Häse, Christoph Kreisbeck, Teresa Tamayo-Mendoza, Lars P. E. Yunker, Jason E. Hein, Alán Aspuru-Guzik

**Affiliations:** 1 Department of Chemistry and Chemical Biology, Harvard University, Cambridge, Massachusetts, United States of America; 2 Department of Chemistry, University of British Columbia, Vancouver, British Columbia, Canada; 3 Department of Chemistry and Computer Science, University of Toronto, Toronto, Ontario, Canada; 4 Vector Institute for Artificial Intelligence, Toronto, Ontario, Canada; 5 Canadian Institute of Advanced Research, Toronto, Ontario, Canada; University of South Carolina, UNITED STATES

## Abstract

The current Edisonian approach to discovery requires up to two decades of fundamental and applied research for materials technologies to reach the market. Such a slow and capital-intensive turnaround calls for disruptive strategies to expedite innovation. Self-driving laboratories have the potential to provide the means to revolutionize experimentation by empowering automation with artificial intelligence to enable autonomous discovery. However, the lack of adequate software solutions significantly impedes the development of self-driving laboratories. In this paper, we make progress towards addressing this challenge, and we propose and develop an implementation of ChemOS; a portable, modular and versatile software package which supplies the structured layers necessary for the deployment and operation of self-driving laboratories. ChemOS facilitates the integration of automated equipment, and it enables remote control of automated laboratories. ChemOS can operate at various degrees of autonomy; from fully unsupervised experimentation to actively including inputs and feedbacks from researchers into the experimentation loop. The flexibility of ChemOS provides a broad range of functionality as demonstrated on five applications, which were executed on different automated equipment, highlighting various aspects of the software package.

## Introduction

Empowering automated infrastructures with artificial intelligence (AI) algorithms has the potential to expedite scientific discovery through autonomous experimentation. [1–4] Inspired by the increasing digitization of science and the rapid expansion of the *Internet of Things* (IoT), the scientific community has begun to design the next generation of research facilities. [5, 6] The concept of autonomous and interconnected robotics platforms with specific tools to tune the properties of materials will enable new fabrication strategies. [3] Such platforms are at the heart of what we refer to as the *self-driving laboratories*. In the self-driving laboratories, AI algorithms continuously learn from experimental results collected through real-time feedback to construct a model to hypothesize about the experimentation at hand. With new measurements, this model is refined to improve recommendations for the next experiment to run. This procedure defines the closed-loop approach. It allows to obtain information-theoretic driven decisions to maximize knowledge acquisition in order to reach a set of predefined goals, such as maximizing the yield of a reaction, reducing the time of an experiment or minimizing reactants usage. As such, this approach differs from combinatorial chemistry [7–10] in an essential aspect; in combinatorial chemistry, experimental campaigns are designed prior to starting the experimentation process, whereas the proposed approach has campaigns that adapt at every closed-loop iteration. Although the premise of autonomous research facilities has recently been analyzed, [3, 11] a portable, modular and versatile software interfacing researchers, robots, and computational tools to orchestrate and facilitate the deployment of these self-driving laboratories is yet to be designed. Such a software package is presented herein, and is referred to as ChemOS.

Automation was pioneered by the industrial sector in search for intensifying chemical and pharmaceutical processes to increase productivity and improve quality. [7, 9, 10, 12–16] During the last decades, several groups have demonstrated the benefits of automated systems on a variety of chemistries, [9, 17–29] and a few laboratory automation software packages have been developed. [30–34] One step further, the integration of design of experiment (DoE) [35–37] to automation emerged as a first strategic approach to experimentation. [38, 39] The first closed-loop approach for adaptive experimentation consisted in grid search-based surveys, which identified the most promising starting points for a subsequent local optimization via different flavors of the simplex algorithm. These hybrid approaches were applied to the optimization of chemical reactions. [10, 40–42] However, parameters evaluated from a grid are correlated, which might result in the omission of important features. In addition, grid search based methods require a substantial number of evaluations to capture relevant phenomena leading, eventually, to discovery. As such, to further streamline the discovery process, the next-generation of autonomous laboratories augments automation with AI algorithms. By refining experimental procedures based on the most recent observations, the AI algorithms ensure to carry out an optimal set of experiments, avoiding the exhaustive and enduring exploration of the complex and high-dimensional application space.

Several groups have already demonstrated the use of autonomous approaches encapsulating modern AI algorithms to a range of applications. One of the first examples of a self-driving laboratory was reported by Maruyama *et al*. on the synthesis of carbon nanotubes. [43] Similar approaches were used to produce Bose-Einstein condensates (BEC), [44] optimize organic synthesis reactions, [45–47] discover multicomponent NiTi-based shape memory alloys, [48] design quantum optics experiments to achieve a certain photonic quantum state, [49] and synthesize and crystallize polyoxometalate clusters. [50] The latter example reports the advantages of such a procedure in terms of accuracy and coverage of the crystallization space by comparing with human-based and random search approaches.

Although the self-driving laboratories appear to be on track to revolutionize the traditional Edisonian approach [51] to experimentation, they require versatile software to be engineered. This often imposes constraints on the development of such autonomous facilities, preventing their full exploitation. In the aforementioned examples, as well as in other applications reported in the past, [17, 52] in-house developed software packages tied to the hardware and to the scientific procedure were used to operate the experimental infrastructure, parse information, and learn from it. Undoubtedly, distinct scientific challenges require distinct and tailored self-driving laboratories. However, a substantial number of processes are common across them, or can be abstracted. Additionally, the significant advances in high-level programming languages, and in computer science, notably in AI, have enabled the design of a flexible software package that addresses the specific needs of autonomous laboratories. As such, it becomes possible to provide the scientific community with a structured software package, consisting of fundamental layers common to any self-driving laboratories. These layers require database management, experiment scheduling, collection of experimental feedback, interaction with different learning procedures, and recommendations of experimental conditions for the robotics platform. In what follows, we present ChemOS, a software package that fulfills these requirements and enables the remote control of robotics. ChemOS has the potential to increase the discovery rate across chemistry and material science and also catalyze the realization of prototype self-driving laboratories.

ChemOS coordinates the overall computational and experimental workflow, monitors experiments, administrates data collection, data storage as well as details about the configurations of the available automated laboratory equipment, potentially distributed across different physical laboratories. One of the crucial components of ChemOS are the various AI algorithms encapsulated in the learning module. Although ChemOS shares the vision of a fully autonomous discovery platform, it allows for an efficient interaction between researchers, AI algorithms, and the robotic hardware. To this end, ChemOS provides various intuitive interfaces for interactions, ranging from simple data exchange via well-defined protocols to natural language processing (NLP).

We demonstrate the performance of ChemOS on five applications, each highlighting different aspects of its implementation. Our findings show the ability of ChemOS to successfully run at a diverse level of autonomy, from fully unsupervised experimentation to actively including the researchers in the closed-loop approach to discovery. Also, we confirm the ability of the AI algorithms to learn experimental procedures on the fly to reach human-defined targets in a minimal number of evaluations on high-dimensional spaces, without prior knowledge. For all tested applications, the same ChemOS core is deployed on several different Unix-like operating systems to operate different (potentially remote) robotic and characterization hardware, with different state-of-the-art AI algorithms, demonstrating the flexibility and modularity of the presented software package. ChemOS is available for download on GitHub at URL: https://github.com/aspuru-guzik-group/ChemOS.

## Results

**Fig 1:**
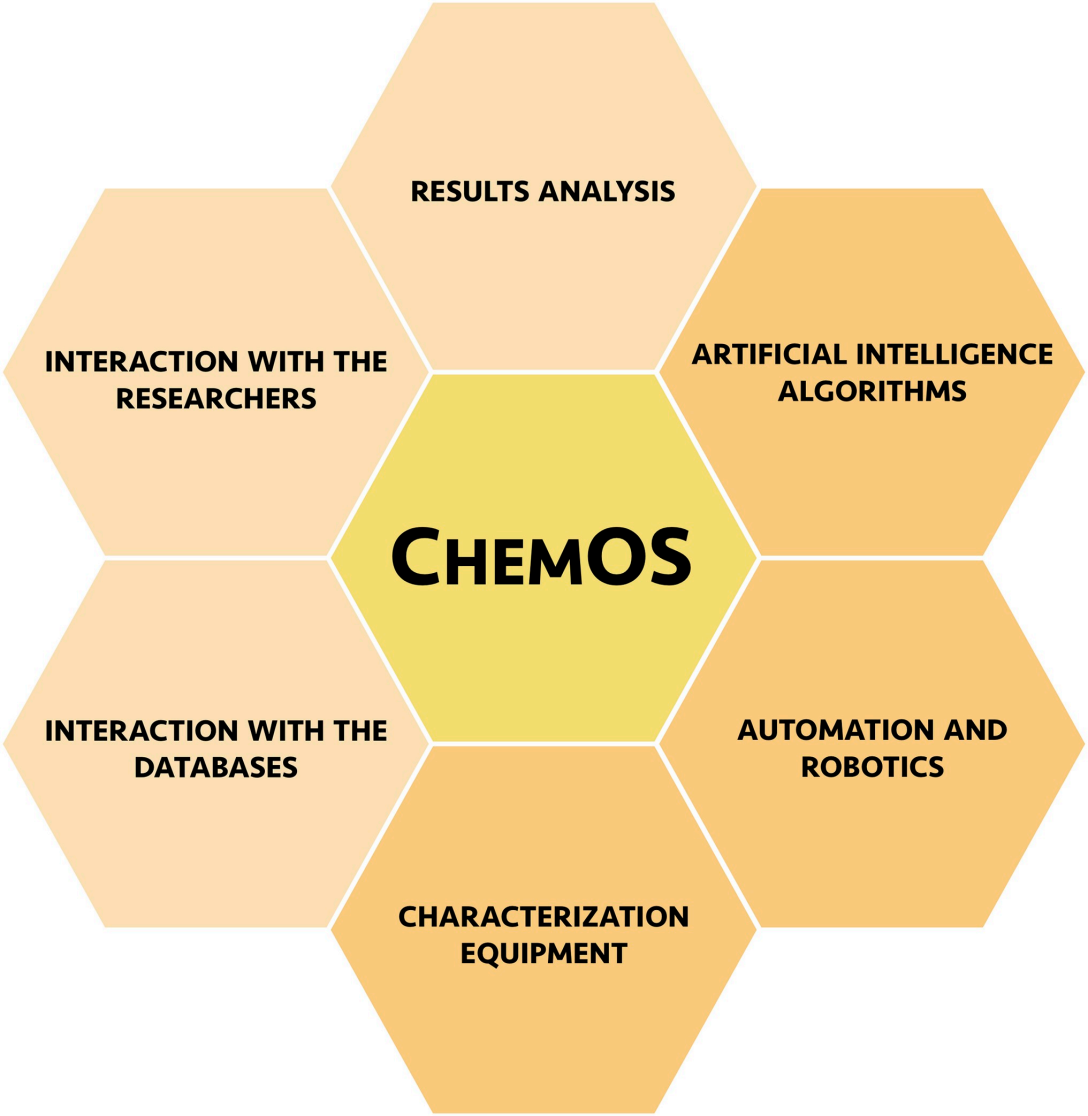
Representation of the modules composing ChemOS. This scheme highlights the modularity and the independence of the six modules, which are (i) global learning procedures, (ii) automated robotic platforms, (iii) characterization equipment, (iv) databases handling and management, (v) intuitive interfaces for researchers, and (vi) online analysis. The central workflow manager, ChemOS, is depicted in yellow. The required modules to reach autonomy in the discovery process are presented in dark orange.

ChemOS follows a modular architecture composed of a central workflow manager and six independent modules (see Fig 1). Three of the modules are required to enable closed-loop experimentation: (i) AI algorithms for experiment planning, (ii) automation and robotics to execute experiments, and (iii) characterization equipment to assess the performance of the conducted experiment. In addition, ChemOS provides modules which improve the practicality of the self-driving laboratories: (iv) databases for long-term data storage, (v) intuitive interactions with researchers, and (vi) online results analysis. The modularity of ChemOS is a crucial element which decouples interdependent tasks. Consequently, ChemOS can be easily extended by incorporating additional modules, or new features specific to an existing module, without interfering with the established workflow. The modularity of ChemOS significantly reduces the obstacles to the development and deployment of the self-driving laboratories. Before demonstrating the performance of ChemOS on applications, we highlight the responsibilities of the individual modules. Detailed descriptions are provided in the supplementary information (see Sec. S1 File).

### Artificial intelligence for experiment planning

The learning module is key to reach autonomy as it designs experimental campaigns for the scientific procedures requested by the researcher. ChemOS abstracts experimental procedures to stochastic response surfaces, which describe the merit of proposed generic experimental conditions with respect to user preferences. The learning module supports various Bayesian machine learning (ML) algorithms for efficient parameter space searches and decision making to recommend conditions for future experiments: Phoenics, [53] SMAC, [54–56] Spearmint, [57, 58] and random search. [59, 60]

### Interaction with automated experimentation hardware and characterization equipment

The robotics and characterization modules provide the mapping between the abstract response surfaces on which the learning procedures operate to the actual scientific procedures. For new applications, the communication protocol and interaction layers allowing to integrate the new hardware and/or characterization equipment within the ChemOS workflow need to be implemented manually. It is important to emphasize that this programming exercise is the only manual step to the deployment, and cannot be automated as the application programming interfaces (APIs) may vary from one automated platform to another. Once integrated, the robotics and characterization modules will automatically relate abstract conditions to hardware specific parameters. Since the robotics and characterization modules contain specificity about the available hardware, the learning module in ChemOS can be agnostic about actual scientific procedures and can thus be applied to different procedures simultaneously.

### Databases for long-term data storage

Long-term storage of experimental data and instructions is facilitated via database-management systems (DBMS). ChemOS features connections to multiple DBMS for flexible data storage at negligible computational overhead (see Sec. S1 File). Efficient closed-loop experimentation is enabled by storing information in distinct databases which allows for parallelized reading and writing operations. All DBs operate on the first-in-first-out (FIFO) principle. Consequently, new requests are queued chronologically, and they are processed as soon as parameters and the robotic hardware are available.

### Interaction with researchers

Rapid advances in AI provide opportunities to redesign the interaction of researchers with experimentation equipment. Approaches such as graphical user interfaces (GUIs) raise the deployment obstacles as researchers need to acquaint themselves with the interface first. ChemOS provides more intuitive interfaces for researchers in the form of a chatbot framework powered by a NLP module. This interface favors the interaction between researchers, the learning module, and the robotic hardware. The NLP module processes new requests, filters and summarizes relevant results and customizes responses based on information specific to the received messages (see Fig 3c). ChemOS connects the NLP module to several common social media platforms and communication services including e-mail exchange, private and public messaging via Twitter, and private messaging via Slack. As such, ChemOS can accommodate the individual preferences of a researcher or a research group. Communication via multiple channels in one session is also supported.

### Online analysis of experimental results

ChemOS features an analysis module to process, summarize and visualize the results obtained from measurements. This enables researchers to quickly perceive the progress of the current experimentation and conceptualize the findings of the self-driving laboratory. ChemOS supports the generation of time traces, runs statistics on repeated experiments, computes higher-level objectives from lower-level experimental observations and visualizes the search space of the experimental procedure. Researchers can request a status report at any time in the course of the experimental procedure. Fig 3c illustrates such a status report requested via Slack.

### Orchestrating experimental procedures with ChemOS

The modular implementation and the decoupling of individual tasks of the closed-loop experimentation process enable ChemOS to orchestrate multiple experimental procedures simultaneously at negligible computational overhead (see Sec. S1 File). Individual experimentation sessions can be implemented by providing detailed information and feature selections for each of the modules in a single configuration file (see Sec. S1 File). The configuration file includes the researchers’ choices of learning procedures and communication channels. Furthermore, it informs ChemOS about the available automated experimentation platforms. Then, ChemOS automatically maps experimental procedures to the available hardware. Consequently, deploying ChemOS to new applications only requires to modify the configuration file. This flexibility allows for an accelerated and simplified deployment of the self-driving laboratories, and it empowers ChemOS to orchestrate numerous experiments for different applications.

### Orchestration of standard laboratory equipment

We suggest a robotic liquid handling platform to produce blends to yield a pre-defined color, pH and density from a set of starting materials. We demonstrate that ChemOS is capable of generating such formulations without human supervision, and that the produced formulations match the pre-defined goals.

**Fig 2:**
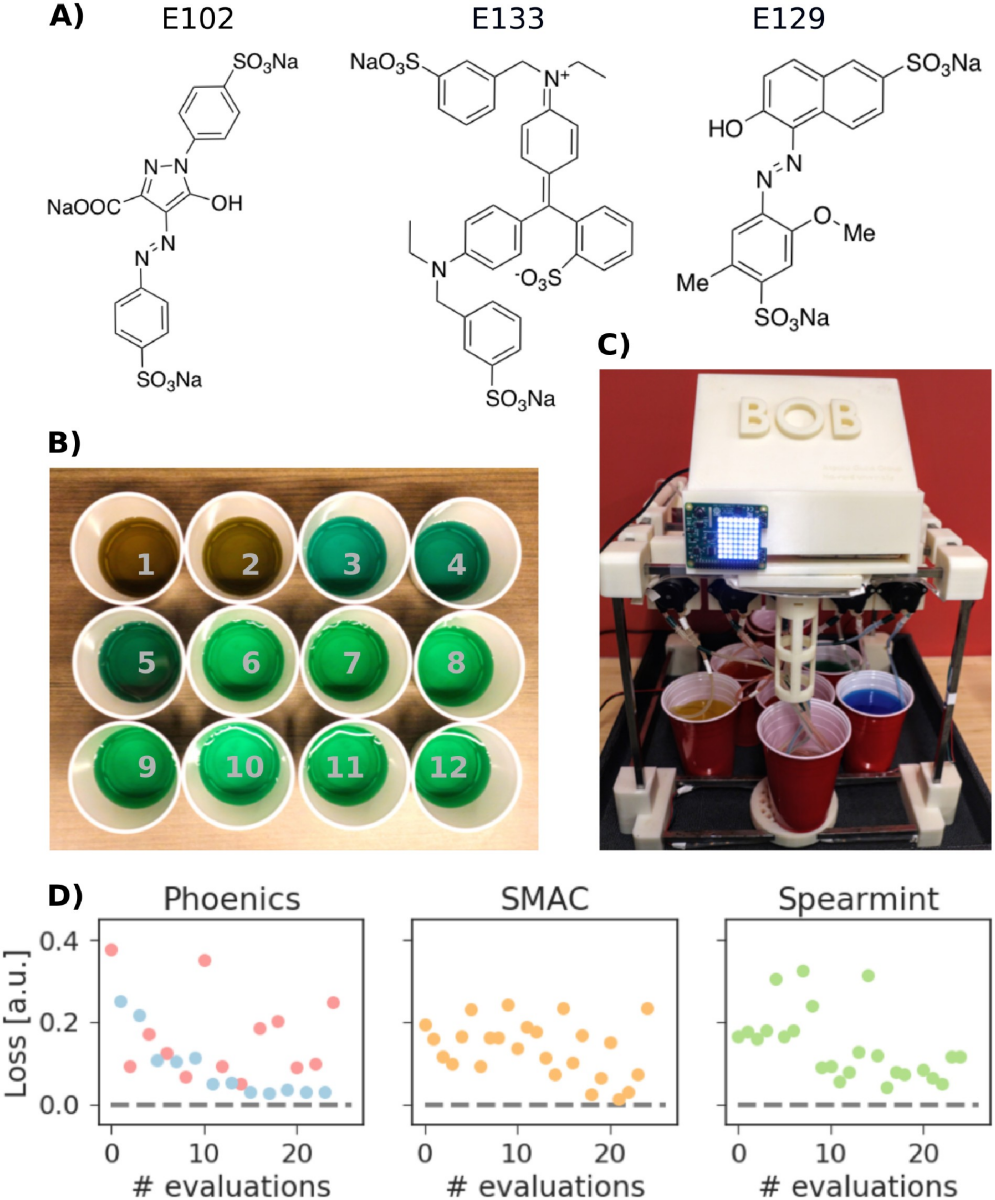
(a) The three dyes used in this experiment: E102 (yellow), E129 (red), and E133 (blue). (b) Picture of the solutions obtained with the 12 exploitation points from Phoenics (c) Picture of the in-house built robot. (d) Maximum norm distance (loss) between the achieved normalized RGB color code and the target RGB color code for the 25 experiments. Each panel corresponds to the learning procedure in-use: Phoenics, SMAC, or spearmint. For the Phoenics algorithm, red denotes a bias towards exploration, and blue a bias towards exploitation. Note that in exploration mode, Phoenics samples parameters to gather knowledge where the algorithms has only limited information. In exploitation mode, however, Phoenics makes the best decision given current information and suggests parameters in the vicinity of the best performing experiment.

**Fig 3:**
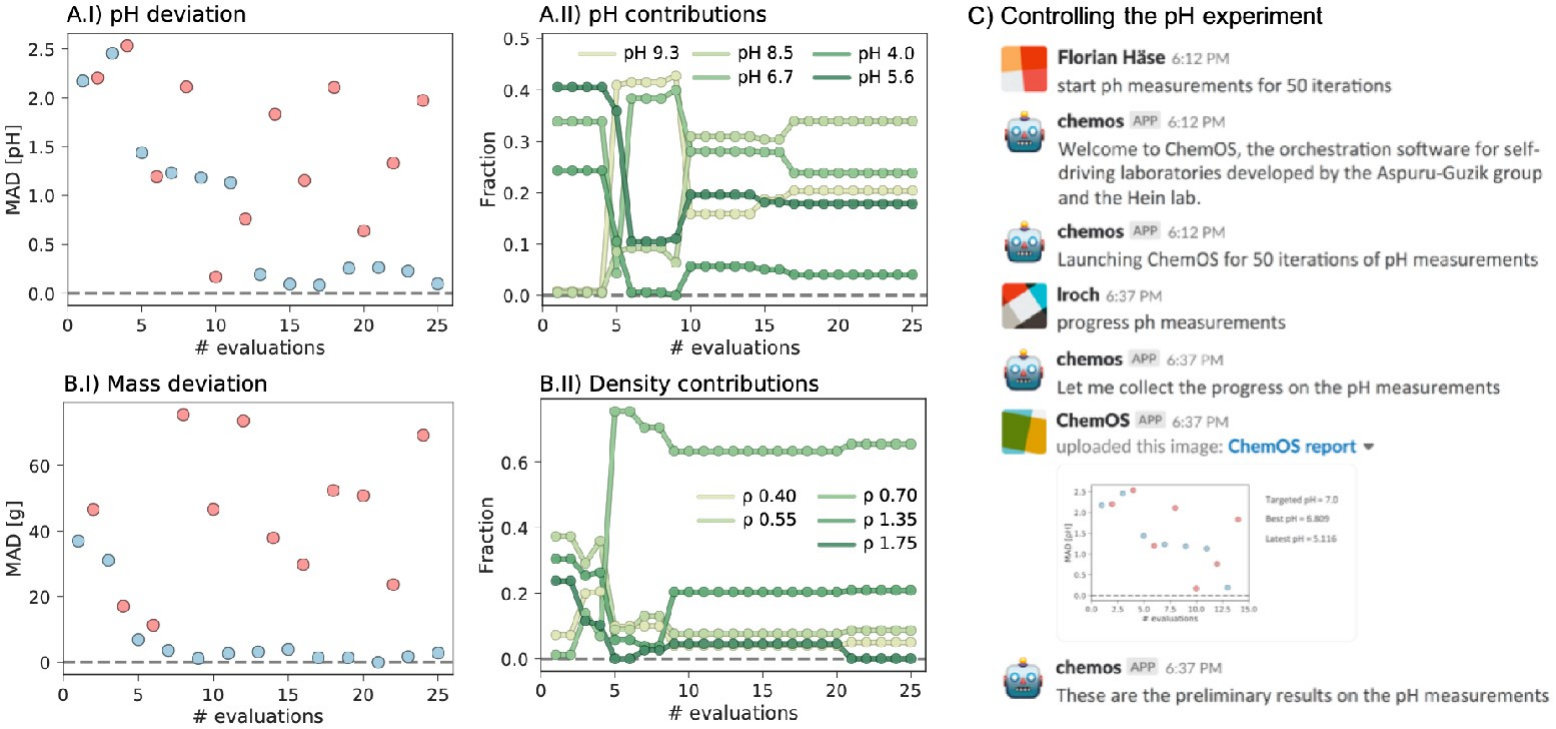
Results from the pH (A) and density (B) experiments. Both the loss (I) and the contribution of the starting materials to the produced mixture (II) are reported. (C) Example of a dialogue between ChemOS and researchers.

The experiments outlined in this section were controlled on and synchronized across three distinct physical platforms: (i) a master platform hosting ChemOS and the learning procedure, (ii) an automated robotic platform operating the liquid handler (see Fig 2c), and (iii) a characterization platform controlling the characterization equipment for each of the experimental procedures (RGB sensor, pH meter, and a precision laboratory balance). The communication between the master platform and the characterization platform is supported via Dropbox, while the liquid handler and the characterization platform communicate via the Secure Copy Protocol (SCP). All communications are supervised and instantiated by ChemOS from the master platform. The experimental loop is initialized remotely by the researchers, as illustrated in Fig 3c. Once the researchers’ request is parsed, ChemOS uses the learning module to recommend a first experiment. The robotics module maps the proposed experiment parameters to the available hardware to execute the experiment. The characterization station measures the properties of interest, which are compared to the target defined by the researcher. Based on this feedback, ChemOS then recommends promising experiments for future evaluations.

#### Learning the color space

This procedure generates a target colored solution from a set of five dyed solutions combining three individual dye molecules at different concentrations (Fig 2) in full autonomy. This first use-case demonstrates the closed-loop approach orchestrated by ChemOS, and highlights the workflow management. ChemOS was instructed to produce a green solution, and could produce the target by mixing red, orange, yellow, green and blue solutions (see Materials and methods for details). Importantly, ChemOS was not provided any information about the initial solutions and was not constrained to the number of solutions to use. As such, ChemOS was free to generate the target green from, for example, choosing only the provided green solution, or mixing the yellow and the blue solution.

We ran this experimental procedure with all three implemented AI algorithms (Phoenics, SMAC and spearmint) for a total of 25 experiments per session. Fig 2d displays the progress of the experimental procedures for each algorithm. The reported *loss* indicates the maximum distance between the normalized RGB codes of the sampled solution mixtures and the normalized RGB code of the target color. The rapid decrease of the loss validates the capability of ChemOS to learn the color space in full autonomy. All employed learning algorithms consistently produce mixtures of green color with RGB codes close to the desired target. An example of colors produced by the mixtures sampled from Phoenics with a bias towards exploitation is displayed in Fig 2b.

This simple experiment illustrates the ability of the learning procedures to suggest routes to reach pre-defined targets which deviate from human intuition. Where a human researcher might favor the green starting solution over a mix of multiple starting solutions to produce a green target, ChemOS suggested a recipe containing 43.6% of the green solution, 19.6% of the yellow solution, 30.0% of the blue solution, and 6.8% the orange solution. This mixture still reproduced the target color indistinguishable by both the human eye and the RGB sensor and presents an unexpected solution to the given task.

#### Learning the pH space

The goal of this procedure is to generate a solution with a desired pH value using five different starting materials with different pH values. The target pH value is defined by the researcher and set to pH = 7.0 in this example. The five starting materials were prepared with potassium hydrogen phthalate (pH = 4.0, and 5.6), mixed phosphate (pH = 6.7) and borax (pH = 8.5, and 9.3). ChemOS used the Phoenics algorithm to produce the desired target pH within 25 experiments. Fig 3a illustrates how the mixtures proposed by the algorithm approach the target pH of 7.0. Panel 3a.I depicts the pH values measured in each experiment, while panel 3a.II shows the best performing composition. No constraints were applied to the number of starting materials to use, as was already the case for the color mixing experiment.

We observe a rapid decrease in the deviation of the produced pH value from the desired target for this five dimensional search space with experiment 10 already yielding a pH of 6.809. The closest experiment to the target is experiment 17 where the pH of the solution was measured to be 7.001. This second illustration showcases the ability of ChemOS to run in full autonomy. It also highlights the ability of the AI algorithm to learn experimental procedures on the fly to reach a human-defined target in a minimal number of evaluations on high-dimensional spaces, without prior knowledge.

#### Learning the density space

In this procedure, ChemOS is provided with five fluids of different densities with the target to produce a blend with a desired target density of 1 g/cm^3^. Like in the previous examples, the densities of the provided fluids (0.40, 0.55, 0.70, 1.35, and 1.75 g/cm^3^) were hidden from ChemOS. ChemOS controlled this procedure with the Phoenics learning algorithm for 25 experiments, following the setup of the pH experiment. Fig 3b reports the deviation of the density of the produced blend from the target density, as well as the contribution of each individual starting material to the blend. Within only nine experiments in this five dimensional space, Phoenics reached the targeted density of 1 g/cm^3^.

In summary, these three examples demonstrate the capacity of AI algorithms to efficiently probe and evolve on high-dimensional parameter spaces while performing at an optimal number of experiments even without prior knowledge. By not supplying AI algorithms with any prior assumptions, the algorithms are enabled to discover solutions which can be beyond the researchers’ expectations. This is of utmost importance when targeting scientific discovery. In fact, observations of unbiased AI algorithms finding creative and unexpected solutions have been made recently in the field of evolutionary computation and artifical life. [61] Finally, these three applications illustrate the closed-loop approach to experimentation implemented in ChemOS. They also highlight the seamless integration of different characterization equipment (RGB sensor, pH meter, and precision laboratory balance) into the ChemOS workflow. The potential of such an approach extends far beyond these applications; we can imagine the potential of ChemOS when bridged to platform design for drug and material discovery.

### Integration of the researcher in the loop to learn the *Tequila Sunrise* space with Bob

We further demonstrate the flexibility and modularity of ChemOS on a procedure for mixing consumable liquids. In this procedure, we modified the robot used for the previously reported applications and named it “Bob”, for “Bayesian Optimized Bartender”. The goal of this procedure is to identify recipes for Tequila Sunrise, which are judged based on the taste and preferences of the researchers. This poses the challenge to increase the level of interaction between researchers and robots, as an active integration of humans into the feedback loop is required. This integration is of utmost importance in processes requiring human approval such as in the pharmaceutical, food, and cosmetics industry. [4]

**Fig 4:**
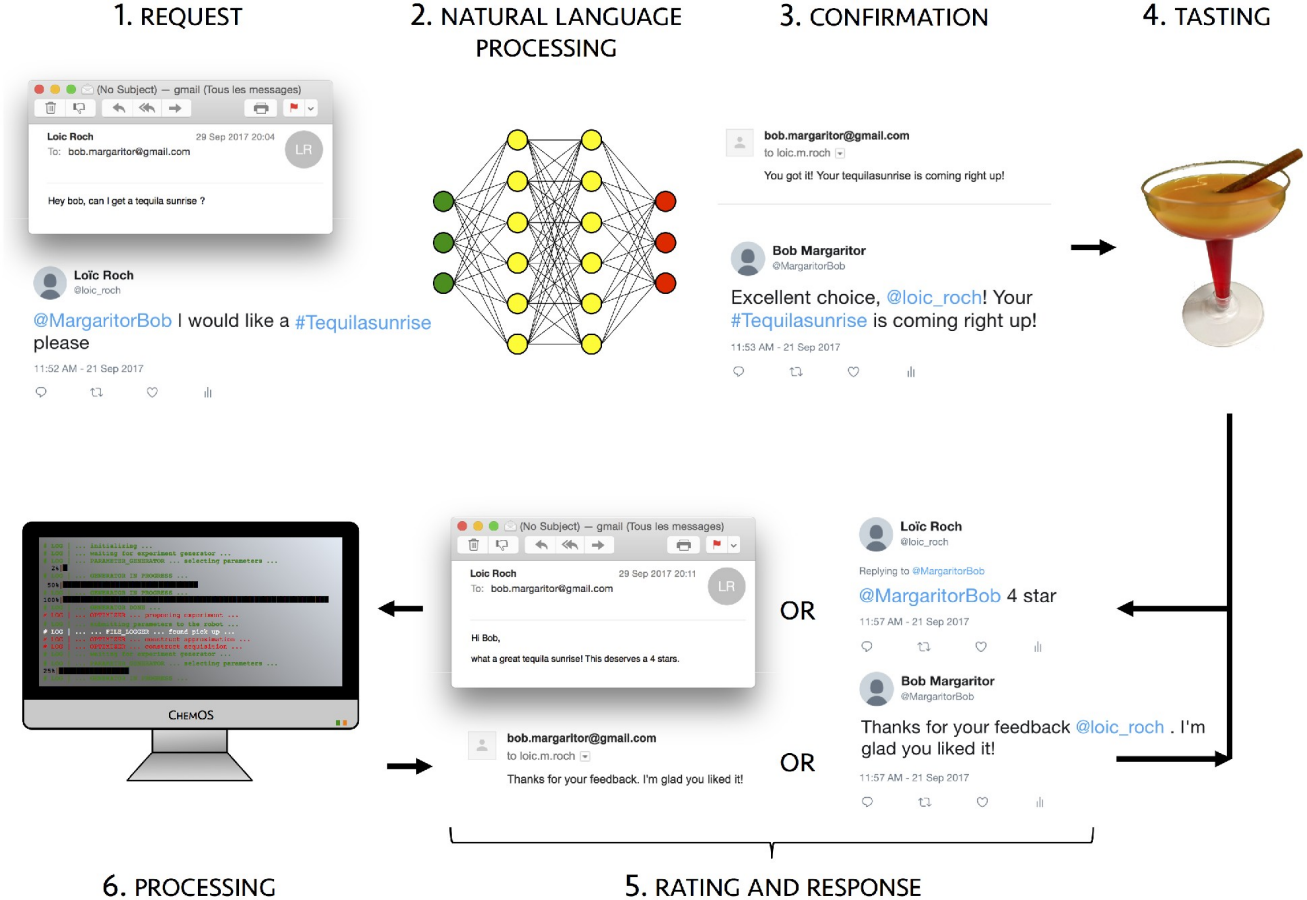
Representation of the ChemOS pipeline while screening the Tequila Sunrise space.

The overall procedure (see Fig 4) starts with a researcher requesting a new cocktail via e-mail or twitter. Requests are processed in the same way as in prior applications. Order confirmations and notifications of completion of the mixing procedure are then sent by ChemOS to the researcher, which serves as the characterization module in the ChemOS cycle. A five-star rating system is used as feedback to assess the taste of the cocktail. As was the case for the color space application, there were no constraints on the ratios of ingredients.

We conducted the experiment with four researchers in a single session, and ChemOS used the Phoenics algorithm. Results on the conducted experiments are reported in the supplementary information (see Sec. S1 File). We note that during the progress of the experimental procedure, researchers gave generally more positive ratings towards the end of the procedure, indicating that the taste of the mixtures improved over time. However, we did not observe a strong preference for certain recipes as in prior applications. The outcome might reflect the subjective nature of the objective function due to the large differences in the taste among the researchers.

This case-study highlights the fact that ChemOS provides a flexible and interactive platform, enabling communication between researchers and robotics solutions at various degrees of autonomy. Importantly, the ChemOS core was not modified to specifically suit this application. Instead, the degree of interaction was increased by simply modifying specifications in the configuration file.

### Autonomous calibration of a remote robotic sampling sequence for direct-inject HPLC analysis

Hereafter we illustrate how ChemOS can be used for remote interactions with distributed automated laboratory systems. We orchestrate an infrastructure capable of unattended sampling. A similar setup was recently used for real-time reaction progress monitoring. [26] The robotics hardware is located in Vancouver, Canada, and controlled by ChemOS in Cambridge, USA. The procedure involves proposing new calibration parameters, running the experiment, analyzing the experimental results and updating parameter candidates.

The goal of the calibration is to find a set of parameters which maximizes the response of the HPLC, namely maximize the amount of drawn sample reaching the detector. The workflow is fully controlled by ChemOS and does not require human intervention. ChemOS is set up to communicate with researchers *via* the command line interface for local execution, and *via* Slack for remote execution. The communication between the master platform and the robotic system is enabled via Dropbox. Each ChemOS session is set up for a total of 100 autonomous experiments over a time period of about seven hours. We further demonstrate the robustness of ChemOS on two sessions accumulating a total of 1400 data points. Details are provided in the supplementary information (Sec. S1 File).

**Fig 5:**
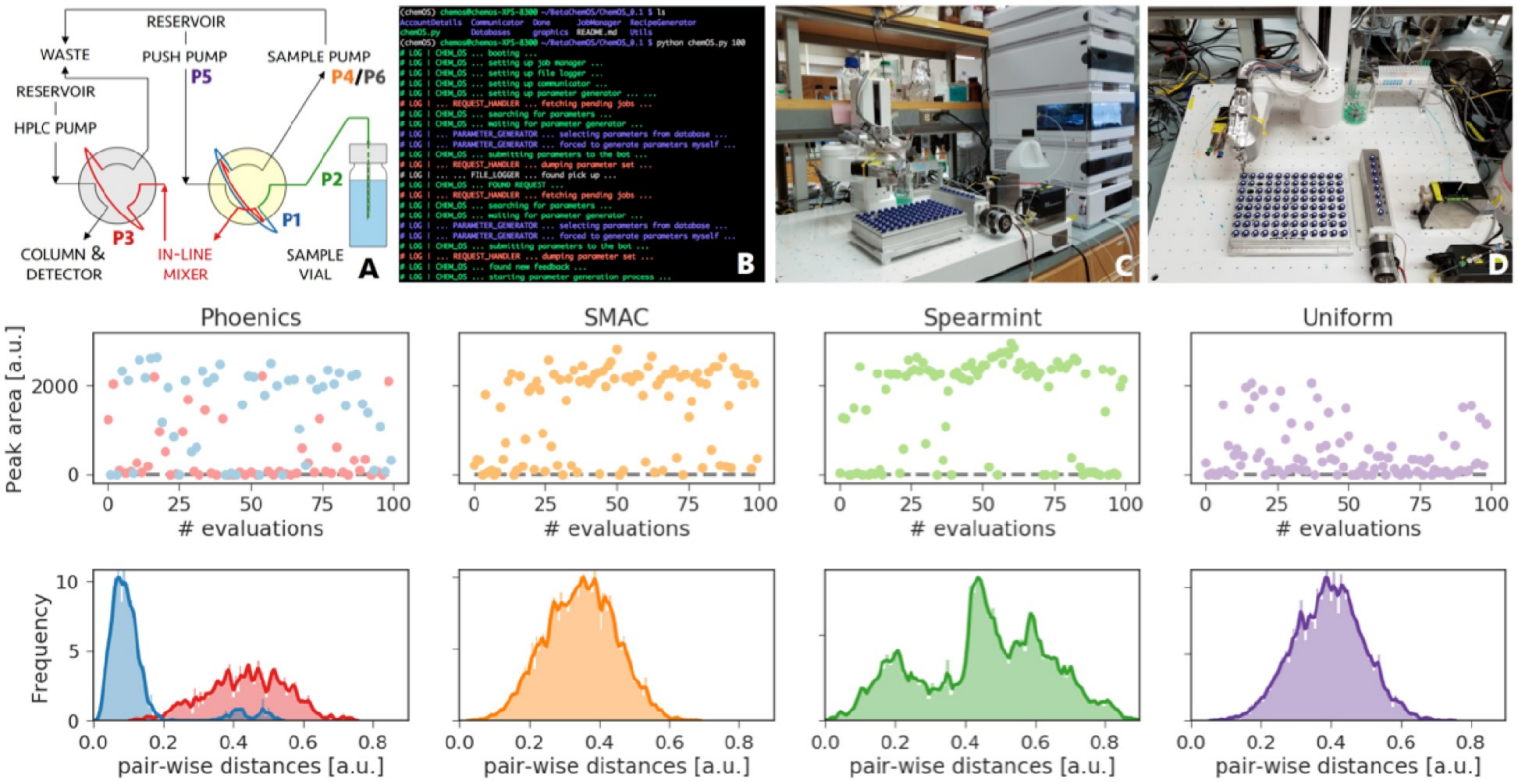
(A) Schematic of the flow path for the sampling sequence used with the N9 robotic platform. The six parameters (P1-P6) are color coded to illustrate the effect they have on the sampling sequence. The yellow shade highlights the arm valve, and the grey shade the HPLC valve. (B) Example of logging messages from ChemOS. (C) Side and (D) top view of the robotic hardware. Lower panels: Results from the autonomous calibration of an HPLC setup maximizing the magnitude of the response. ChemOS performed autocalibration with four different learning procedures (Phoenics, SMAC, Spearmint and Uniform). Upper panels display the achieved peak areas, i.e. magnitudes of response. Lower panels display the distributions of pair-wise distances between sampled parameter points computed with the L2 norm. For the Phoenics algorithm, red denotes exploration, and blue exploitation.

The experimental arrangement is illustrated in Fig 5a. A robotic arm (N9 from North Robotics) with an integrated sampling needle is used to draw a sample of 1,3,5-trimethoxybenzene in MeCN from one of the vials in a 96-well tray. The sample is then passed through a sample loop, inline mixer, and injection loop and is finally analyzed by an HPLC. Peak areas of the chromatogram determine the amount of target compound, which was delivered to the HPLC. The entire setup is controlled by six independent parameters determining sample draws, wait times and push rates. A detailed description of the individual parameters as well as their influence on the peak response are provided in the supplementary information (see Sec. S1 File).

We demonstrate the autonomous execution of the autocalibration by ChemOS with the four learning procedures: Phoenics, SMAC, spearmint, and random search. The results of each autocalibration run with 100 individual experiments are depicted in Fig 5. The upper panels display the peak areas achieved during the execution of individual experiments with the different learning procedures. The uniform random searches of the parameter space represent an uninformed exploration of the parameter space as acquired knowledge is not taken into account when proposing new parameter points. We observe that all implemented optimization algorithms (Phoenics, SMAC and spearmint) quickly find parameter points which yield peak areas larger than those encountered in the random search. The lower panels of Fig 5 depict the parameter distance distributions computed from all possible parameter pairs sampled in each ChemOS run. While uniform random search appears to yield a unimodal distance distribution, the optimization algorithms show tendencies of favoring broader distance distributions with more parameter points at both smaller and larger distances to other parameter points. We interpret this deviation in the distance distributions as the signature of the optimization algorithms used, in which the acquisition function emphasizes either exploitation of the acquired data (small distances) or exploration of the parameter space (large distances).

Since repeated executions of individual experiments are time and resource intensive, we assess the performances of individual experiment planning strategies in detail with an emulator constructed to reproduce the experimental surface. Specifically, we train a Bayesian neural network (BNN) to reproduce the response of the HPLC for any given parameter point. This approach has already been demonstrated in the context of self-driving laboratories. [62] Details of the BNN are reported in the supplementary information (see Sec. S1 File).

**Fig 6:**
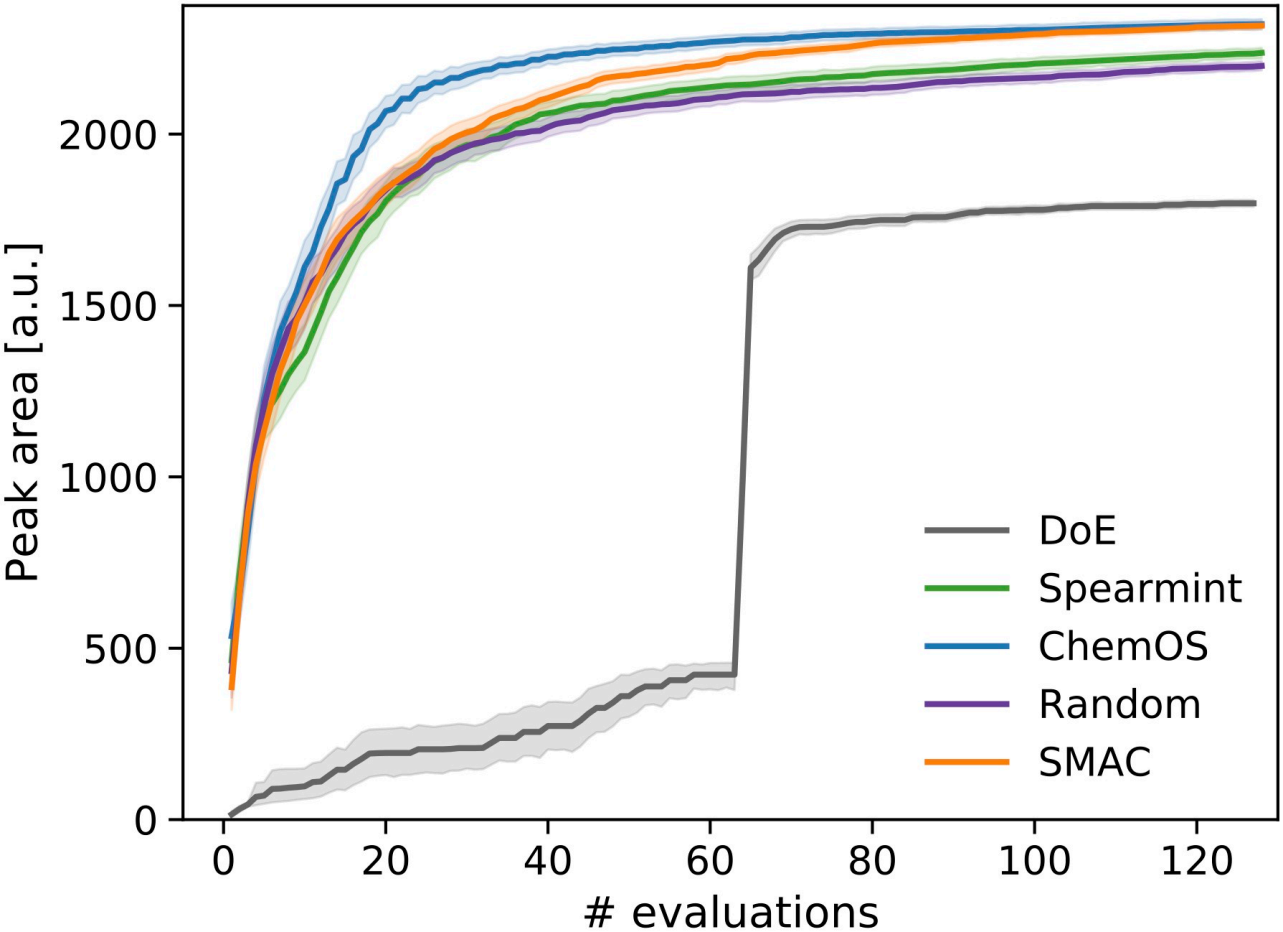
Average performance of the experiment planning strategies leveraged by ChemOS.

The results of the emulator benchmarks averaged over 200 independent executions are illustrated in Fig 6). In addition to the already mentioned experiment planning strategies, we probed the performance of a design of experiment (DoE) approach, where a full factorial grid is used initially to probe the search space and a second, refined full factorial grid is constructed based on the initial responses for an in-depth analysis of a subregion of the search space. We find that DoE is outperformed by the random search strategy, which can be attributed to the high-dimensional nature of the search space. In addition, spearmint displays a comparable performance. Only SMAC and Phoenics demonstrate significantly better average performance.

With this application, we showed that ChemOS can control remote facilities, and that it enables to accelerate process optimization by augmenting automated systems with AI to yield autonomous scientific procedures.

## Conclusion

We introduced ChemOS, a transferable, flexible and versatile software package. ChemOS is a framework, which supplies all the layers needed to control and orchestrate distributed autonomous laboratories. The five applications reported herein highlight this ability and ease to deploy ChemOS and to control different applications with a variety of laboratory equipment and automated solutions by only editing the configuration file. The functional design of ChemOS and its modular structure, in which each module is responsible to fulfill specific tasks, allows for the global control of complex heterogeneous automation platforms. What is more, the flexible architecture enables to update modules independently and facilitates the addition of new features which reduces the obstacles to the deployment of self-driving laboratories.

ChemOS includes state-of-the-art AI algorithms for experiment planning in its learning module. This module is the key component to reach autonomy. Also, to facilitate the researcher-robot interaction, we supplemented ChemOS with an NLP module in a chatbot framework. This module, based on a neural network, interfaces with several common social media platforms and communication services. This module enables the researchers to trigger new experiment from a distance, or to send commands and instructions at any point in the course of the ChemOS cycle. This includes requests for new experiments, status updates, or feedback in plain text messages. Finally, due to parallelization techniques, the negligible computing overhead rising from function queries, database requests and data parsing ensures a maximized experimentation throughput.

The current version of ChemOS is the beginning of a comprehensive software package for controlling autonomous laboratory systems. We believe that ChemOS has the potential to follow the path paved by the Robot Operating System (ROS), [63, 64] to become the standard to power self-driving laboratories. The results shown in this manuscript are the first steps towards a global operating system for distributed automated laboratories. Similar to supercomputer centers, which give researchers easy access to compute infrastructure, we envision ChemOS to democratize autonomous discovery. With the access to such laboratories, more research groups could join the evolution of experimentation and could contribute to advances in a large spectrum of technologies at an accelerated rate.

## Materials and methods

### Color, pH, density and drink experiments

The robot was assembled on a frame of aluminum rods and 3D-printed pieces. Note that the 3D model and stl files were downloaded from GitHub [https://github.com/ytham/barmixvah, author: Yu Jiang Tham], in July 2017. The pumping system was built with a Raspberry Pi 3 Model B as a microcontroller, a power supply, and a set of eight DC peristaltic pumps of 12V, each connected to a 5V relay. Within this arrangement, the relays were controlled by the Python library GPIO. We used SCP as the communication protocol between the Raspberry Pi and ChemOS, which was deployed on a Mint environment (Ubuntu based operating system). The low-level programs controlling the pumps and the RGB sensor, the pH meter, and the scale were written in Python. The measurements obtained by the pH probe (pH probe and circuit by Atlas Scientific), RGB sensor (EZO-RGB by Altas Scientific.), and the scale (Mettler Toledo MS303S) were read on a PC by a serial port with the Python library *serial*, *pylibftdi*, *serial_device2*, respectively. The synchronization between the PC and ChemOS was performed via Dropbox.

The learning algorithms generate five values on the [0, 1] interval. ChemOS formats these five values into an eight dimensional array, according to the specific experimental layout used. Once transmitted to the robot, this eight-value array is re-scaled based on a previous calibration of each individual pump. Then, the vector is scaled to the total volume to be drawn, i.e. the sum of the five initial solutions. Finally, the RGB sensor measures the color of the solution. ChemOS receives only the maximum norm distance between the target normalized RGB vector and the measure RGB (also normalized). The latter is minimized during the ChemOS run.

Five initial solutions with the following normalized RGB codes are provided: yellow (RGB = [0.40,0.41,0.19]), red (RGB = [0.70,0.17,0.13]), orange (RGB = [0.57,0.26,0.17]), blue (RGB = [0.06,0.36,0.58]), and green (RGB = [0.16,0.56,0.28]). The provided green was chosen as the target color for this procedure. Through the robotics module, ChemOS automatically maps the proposed experimental parameters to the order of the pumps on the robotic hardware. Importantly, ChemOS was not constrained to the number of solutions to use. For example, green solutions can be produced by choosing only the provided green mixture, or mixing the separate initial yellow and blue solutions. Details on the procedure are provided in Sec. A. Identical setups were used for the pH and the density experiments.

This robot was slightly modified to yield the Bayesian Optimized Bartender, Bob. ChemOS communicates with the researcher using Twitter and Gmail. It processes messages through the NLP module, which classifies incoming messages as request or feedback. A set of digital LED strips regulated with the I2C protocol and with the Python library *fcntl* was used to inform the researchers upon completion of a process.

### Autocalibration experiment

The robotic setup was built on a North Robotics N9 platform, paired with an Agilent 1260 Infinite series HPLC. The sample used was 10 mM 1,3,5-trimethoxybenzene in MeCN. The sampling sequence involves first the drawing sample through a needle, and then a sample loop installed into a Rheyodyne 2-way 6-port selection valve. The valve is then switched, and the diluent solvent is pushed through the sample loop and an in-line mixer to a second loop in another 2-way 6-port selection valve. This second valve is then switched to be in line with the HPLC pump and column and the HPLC acquisition is triggered. A full cycle of operation involves retrieving a set of parameters from ChemOS, executing a sampling sequence, rinsing the sampling needle and the push line, retrieval of the integration from the HPLC trace, and return of the integrated value to ChemOS. Dropbox was used as the communication protocol.

The control software for the robot was written in Python, which enabled the variation of several parameters of the sampling sequence. The parameters from ChemOS are passed to a sampling sequence function, which incorporates those parameters into a sequence of arm movements, pump manipulations, and valve switches. The commands in that sequence are function calls of Python class instances specific to the physical object being manipulated. Pump and valve switch commands are then relayed through the robot to those components. Arm motion commands are calculated and determined dynamically from the target location (sample or rinse vial) prior to being passed to the robot. The parameter values were obtained as normalized values from ChemOS, and were scaled to the appropriate ranges. The scalars were user-defined to restrict the values within reasonable ranges accessible by the hardware.

## Supporting information

S1 File (PDF)
