# Supplementary material for: ChemOS: An orchestration software to democratize autonomous discovery
Source: PLoS One. 2020 Apr 16;15(4):e0229862. doi: 10.1371/journal.pone.0229862 (PMC7161969; doi:10.1371/journal.pone.0229862)
Supplement: S1 File — (PDF) [file pone.0229862.s001.pdf]

ChemOS: an orchestration software to democratize autonomous discovery
Supplementary Information

Loïc M. Roch,[1,*] Florian Häse,[1,†] Christoph Kreisbeck,[1] Teresa Tamayo-Mendoza,[1]
Lars P. E. Yunker,[2] Jason E. Hein,[2] and Alán Aspuru-Guzik[1,3,4,5,‡]

[1]Department of Chemistry and Chemical Biology, Harvard University, Cambridge, Massachusetts, 02138 USA
[2]Department of Chemistry, University of British Columbia, Vancouver, British Columbia V6T 1Z1, Canada
[3]Department of Chemistry and Department of Computer Science, University of Toronto, Toronto, Ontario M5S 3H6, Canada
[4]Vector Institute for Artificial Intelligence, Toronto, Ontario M5S 1M1, Canada
[5]Senior Fellow, Canadian Institute of Advanced Research, Toronto, Ontario M5G 1Z8, Canada

(Dated: February 10, 2019)

Contents

S.1. Details on the architecture of ChemOS 2
S.1.1. Artificial intelligence for experiment planning 2
S.1.2. Automated experimentation hardware 2
S.1.3. Databases for long-term data storage 3
S.1.4. Natural language processing in a chatbot sframework 3
S.1.5. Workflow of ChemOS 4
S.1.6. Runtime performance of ChemOS 4

S.2. Configuration of distinct experimental procedures with ChemOS 5

S.3. Details on the cocktail experiments 9

S.4. Extensive calibration of a robotic sequence for direct-inject HPLC analysis 9

References 10

*These authors contributed equally; Electronic address: loic.m.roch@gmail.com
†These authors contributed equally; Electronic address: hase.florian@gmail.com
‡Electronic address: alan@aspuru.com

S.1. DETAILS ON THE ARCHITECTURE OF CHEMOS

ChemOS is designed with a modular architecture to orchestrate unsupervised experimentation on heterogeneous experimentation platforms for autonomous discovery. The core module of ChemOS serves as a workflow manager. As such, it connects to all modules, controls the flow of information between modules and schedules tasks to be executed on each module. Tasks are scheduled asynchronously to parallelize experiment planning and experiment execution, which maximizes the overall experimentation throughput.

S.1.1. Artificial intelligence for experiment planning

ChemOS uses artificial intelligence (AI) algorithms to design experiments without human input or guidance. AI algorithms speculate about the outcomes of individual experiments based on all previously conducted experiments. ChemOS interfaces to four different strategies for experiment planning: Phoenics,[1] SMAC,[2–4] Spearmint,[5,6] and random seach.[7,8]

Phoenics is a universal deep Bayesian optimizer developed by Häse *et al.* for global optimization.[1] It is a probabilistic algorithm, which combines aspects from Bayesian optimization with ideas from Bayesian kernel density estimation. As a consequence, Phoenics shows a favorable linear scaling with the number of parameters as well as the number of observations. Phoenics was shown to be particularly well-suited for automated and parallel experimentation as it can propose parameter points with sampling behaviors ranging from full exploration to full exploitation of the previously collected experimental feedback. This is enabled by introducing a sampling parameter in the acquisition function. As a result, with a single (additional) observation, multiple new experimental conditions can be suggested in batches, maximizing parallelization.

SMAC is a Bayesian optimization package based on random forest (RF) models.[2–4,9] RFs are computationally inexpensive regression models with a favorable linearithmic scaling with the number of observations and a linear scaling with the number of parameters. SMAC features a very efficient implementation of RF models, which keeps the computational cost of the optimization procedure to a minimum. However, the model uncertainty needs to be estimated empirically.

Spearmint is a Bayesian optimization package based on Gaussian processes (GPs).[5,6,10–12] GPs provide a flexible way of finding analytic approximations to the objective function based on normal distributions associated with every parameter point. Nonetheless, GP based optimization scales cubically with the number of observations. With this limitation, GP based optimization is typically applied to relatively low dimensional problems for which the optimum can be found in relatively few evaluations.

Random search refers to the exploration of a parameter space via uniform sampling of all parameters. Because this procedure only makes uninformed decisions, and does not memorize observations, it can be used to explore the parameter space in an unbiased, and uncorrelated fashion.[7,8] Random search finds usage in benchmarking performance and detecting errors and misbehavior of the robotic hardware.

S.1.2. Automated experimentation hardware

ChemOS is capable of interfacing to heterogeneous automated experimentation hardware. The robotics and characterization modules within ChemOS provide the required level of abstraction for automated platforms which enables the flexible integration of diverse hardware into the closed-loop approach to experimentation. High-level experimentation instructions defined in the scientific procedure are processed by the robotics and characterization modules into low-level elementary operations, which can be executed on the robotic hardware. High-level instructions include researcher-oriented keywords commonly used in scientific procedures, e.g. *mixing*, *heating*, *sample drawing.* In contrast, low-level instructions define a set of elementary machine-oriented operations, executed by the robotic hardware to perform a single high-level operation. Such a set of low-level operations could consist in encoding the movement of a robotic arm, operating pumps, or pre-processing experimental results. Evidently, low-level instructions are specific to particular robotics and characterization hardware. Nevertheless, the robotics and characterization modules provide bridges which simplify the integration of new robotic hardware into ChemOS without affecting or

requiring changes the other modules.

S.1.3. Databases for long-term data storage

Self-driving laboratories need to monitor every aspect about the experimentation process in order to implement and control the closed-loop approach to experimentation. As such, they provide the unique opportunity to collect experimental information for long-term storage. In principle, this information can then be used to identify general trends between experimental conditions and the outcomes of certain experiments, and to formulate emerging scientific concepts based on these findings.

Long-term storage of information in ChemOS is facilitated via database-management systems (DBMS). ChemOS features a facade connected to multiple adapters, which interact with the APIs of different DBMS. Currently, we support SQLite, a widely used relational database management system embedded into the end program. Nevertheless, we provide interfaces for the integration of other types of DBMS in ChemOS.

ChemOS stores information in four distinct databases (DBs, see Fig. S.2), which serve specific purposes essential to an efficient workflow within ChemOS. All request entries are stored in the request DB, which contains unique identifiers for the scientific procedure associated with the experiment, and a unique key for the experiment itself. The parameter DB contains parameters for the experiment at hand with an indication flagging their previous usage, along with the identifier of the scientific procedure. The robot DB contains robot-specific information, such as the communication protocol, hardware configurations, and status. Finally, experimental results are stored in the feedback DB in addition to information on the scientific procedure and parameters used for a specific experiment.

All DBs operate on the first-in-first-out (FIFO) principle. Consequently, new requests are queued chronologically, and they are processed as soon as parameters and the robotic hardware are available. ChemOS automatically matches generated experimental parameters and qualified robotic hardware to a given request such that monitoring multiple distinct scientific procedures on different platforms with a single instance of ChemOS is possible.

S.1.4. Natural language processing in a chatbot sframework

ChemOS provides an intuitive interaction between researchers, the robotic hardware and AI algorithms. For this purpose, we supplied ChemOS with a natural language processing (NLP) module in a chatbot framework. The chatbot model is based on simple text classification *via* a neural network. The text classifier is used to determine the type of a received message from a set of predefined categories, for which multiple different valid responses are already defined. Once the type of the received message is determined by the neural network classifier, one of the defined associated responses is selected randomly and sent back to the researcher.

The chatbot framework is constructed from conversational intents defined in the Javascript Object Notation (JSON) format. The defined intents are processed using the *natural language toolkit* (NLTK)[13] in Python, and transformed to bag-of-words arrays for processing in TensorFlow.[14] A multi-layer perceptron is then trained on the defined conversational intents to map given inputs, i.e. plain text, to conversational classes, for which a set of responses is predefined. Given a newly received message, the neural network determines the message category, and a response is sampled from the set of responses predefined for this category. Examples of dialogues between the researchers and ChemOS are reported in the main text.

The neural network classifier is implemented in TensorFlow, version 1.3.0, via the tflearn toolkit, version 0.3.2.[15] We use the Natural Language Toolkit (NLTK), version 3.2.5, to tokenize words, from which we then construct a bag-of-words model. This bag-of-words representation serves as input for the neural network classifier. The classifier was trained on a set of predefined patterns, providing examples for inputs of particular categories. We found good prediction accuracies for a neural network classifier with three layers and eight neurons per layer. Layers are connected via sigmoid activation functions except for the last layer, which uses the softmax activation for classification. The neural network was trained for a total of 10^4 epochs with the Adam optimizer,[16] and an initial learning rate of 10^{-2}.

During the training procedure, we found that the classification capabilities of the trained neural network classifier can be improved significantly by providing a more informative training set, i.e. more examples for inputs of particular categories. We could increase the classification accuracy much more by providing more meaningful examples than by adapting the network architecture of the training strategy.

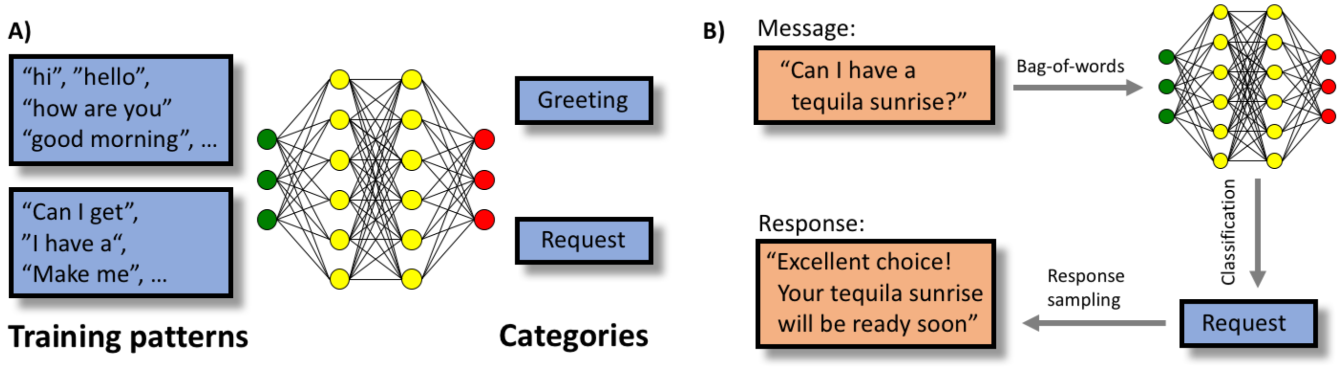

FIG. S.1: Setup and workflow of the natural language processing module in ChemOS. A) A neural network classifier is trained on a set of natural language examples in bag-of-words representations associated with a specific category. B) A previously unseen received message is processed by the trained neural network and classified as one of the defined categories. A response is sampled from the set of predefined responses associated with the classified category.

Fig. S.1 summarizes the setup and the workflow of the natural language processing workflow implemented in ChemOS. The neural network classifier is trained on a set of pattern associated with a particular category before launching a ChemOS instance. When starting a new ChemOS instance the trained neural network classifier is loaded from disk. Newly received messages are transformed into bag-of-words representations and processed by the neural network to determine the category. Once the category of the message has been identified, a response is sampled from the set of responses associated with this category. ChemOS supports the customization of responses based on information specific to the received message. This is achieved by using placeholders in the predefined responses sampled from the neural network classification. At a later stage, the response is replaced with the associated specific information by ChemOS before sending it to the researcher.

S.1.5. Workflow of ChemOS

The general workflow of ChemOS is illustrated in Fig. S.2. During the initialization phase of ChemOS, internal modules are created based on information parsed from a general configuration file. These internal modules comprise (i) the "BotManager", which facilitates communication between ChemOS and both the robotic and the characterization hardware, (ii) the "Communicator", which enables ChemOS to communicate with researchers, (iii) the "ParamGenerator", which controls the learning procedures for proposing new parameters, (iv) the "RequestHandler", which manages new experiment requests, and (v) the "FeedbackHandler", which manages newly received experimental feedback. Then, ChemOS creates the DBs, checks communication protocols and automatically determines which of the defined robots can host the declared experimental procedures. In this process, an internal look-up table is created to accelerate the submission of experiments to the robotic hardware.

S.1.6. Runtime performance of ChemOS

To exploit the full potential of ChemOS, the actions executed at each step throughout the workflow must be parallelized. This not only reduces overhead, but also enables to run multiple experiments at the same time, on different robotics platforms while refining the set of experimental parameters via the learning modules. For example, in a case where the experimental execution of the robotic hardware is time-consuming, the runtime of a single ChemOS cycle is limited by the robotic execution, as learning procedures are executed and monitored in parallel. Such an approach ensures a maximized throughput. In fact, ChemOS is designed to minimize the overall runtime

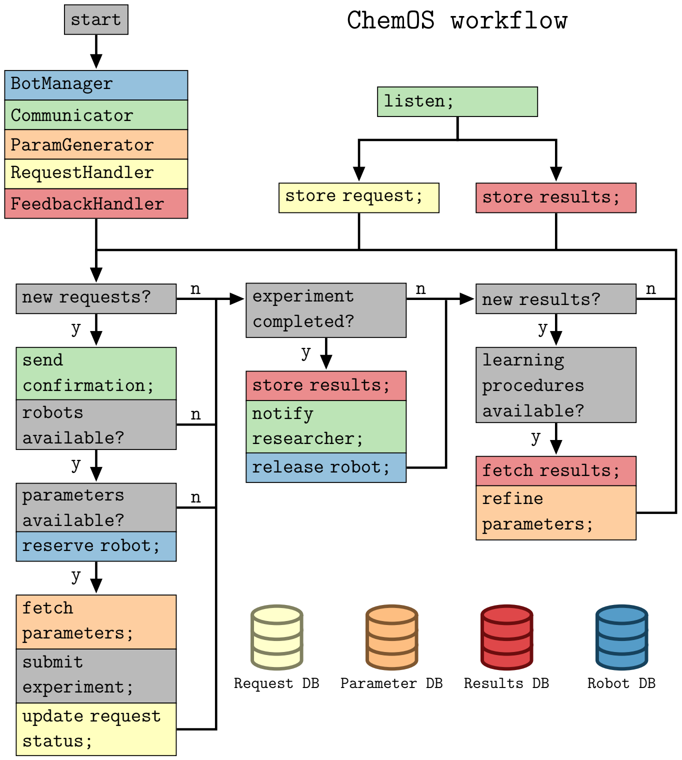

FIG. S.2: Detailed workflow diagram of ChemOS. A new ChemOS session starts with the initialization of five color-coded modules. During one cycle, ChemOS checks for new requests, completed experiments and new experimental results. Each of these events triggers a set of actions. While traversing the loop, ChemOS accepts new requests for new experiments. All information is stored in the corresponding databases.

of the workflow with parallelization techniques. It reduces the wait times of computational resources for extensive parameter generation, and the experiment performed by robotic hardware. This is achieved by executing and monitoring their corresponding modules in separate threads.

The parallel execution of parameter generation and experiment preparation is enabled with the implementation of the parameter DB. Instead of calling the learning module to generate parameters when a request is received, ChemOS looks for an unused set of parameters generated at a previous iteration cycle. The generation of new parameter sets is triggered independently from the requests, as soon as experimental results are received from the robots. Therefore, the parameter DB always contains the most recent parameters.

We suggest the following benchmark test to assess the overhead of the central workflow manager: parameters points are proposed via the random search sampling, and are processed by a virtual robot. The runtime of both the computation and the (virtual) experimentation is set to 4 s. Different scenarios are evaluated, in which execution times for the learning procedure and the robot execution are delayed by 6 s. Fig. S.3 presents four scenarios and compares ChemOS runtime per cycle to the time spent on running the learning procedure and the robotic execution. The reported runtimes were averaged over 100 independent runs.

We observe that the runtime of a single ChemOS cycle is mostly determined by the slower process of generating new parameters and running an experiment. In other words, function queries, database requests and data parsing yield a negligible computational overhead. Hence, the interleaving of learning procedures and experimental execution implemented in ChemOS is an effective design to keep the overall runtime of a ChemOS cycle to a minimum.

S.2. CONFIGURATION OF DISTINCT EXPERIMENTAL PROCEDURES WITH CHEMOS

ChemOS was used to orchestrate a variety of different experimental procedures, e.g. learning the color space, pH space, or density space. All of these experimental procedures were executed with the same core implementation of

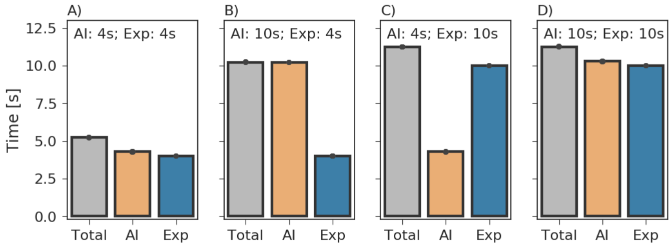

FIG. S.3: Runtimes of full ChemOS cycles compared to the times spent on running an instance of the learning procedure, and evaluating an experiment on the robotic hardware. We display average results with the standard deviation taken from 100 repetitions of the same ChemOS run. Panel (A) shows the scenario where both the learning procedure and the experimental evaluation last 4 s. In panels (B)-(D), an additional delay of 6 s was introduced to (B) only the learning procedure, (C) only the experimental evaluation, and (D) both the experimental evaluation and the learning procedure.

ChemOS. Details specific to each of the experimental procedures, such as available robotic hardware or experimental conditions to refine, were provided via intuitive configuration files. The configuration files include all information needed to orchestrate an experimental procedure in full autonomy, and thus contribute to the flexiblity and simplified deployment of ChemOS.

```
# CONFIGURATION FOR LEARNING THE COLOR SPACE

_SETTINGS = {
# select the learning procedure
’algorithm’: {’name’: ’phoenics’, ’random_seed’: 100691
              ’num_batches’: 1, ’batch_size’: 2,},

# declare file locations for databases
’bot_database’:     {’path’: ’bots.db’,
                     ’database_type’: ’sqlite’},
’param_database’:   {’path’: ’parameters.db’,
                     ’database_type’: ’sqlite’},
’request_database’: {’path’: ’requests.db’,
                     ’database_type’: ’sqlite’},
’results_database’: {’path’: ’results.db’,
                     ’database_type’: ’sqlite’},
’feedback_database’: {’path’: ’feedback.db’,
                      ’database_type’: ’sqlite’},

’communicators’: [  # declare communicators used for this session
    {’type’: ’slack’, ’author’: ’<SLACK_ID>’},
    {’type’: ’gmail’, ’login’: ’<E_MAIL>’, ’password’: ’<PASSWORD>’}],

’experiments’: [    # declare experimental procedures
    # choose unique name, define meta-information
    {’name’: ’color-mixing’, ’repetitions’: 3, ’max_iter’: 25,
     ’description’: ’Create a solution with a target color’
     # define experimental conditions for procedure
     ’variables’:  [
        {’name’: ’red’,    ’type’: ’float’, ’low’: 0.0, ’high’: 1.0, ’size’: 1},
        {’name’: ’orange’, ’type’: ’float’, ’low’: 0.0, ’high’: 1.0, ’size’: 1},
        {’name’: ’yellow’, ’type’: ’float’, ’low’: 0.0, ’high’: 1.0, ’size’: 1},
        {’name’: ’green’,  ’type’: ’float’, ’low’: 0.0, ’high’: 1.0, ’size’: 1},
        {’name’: ’blue’,   ’type’: ’float’, ’low’: 0.0, ’high’: 1.0, ’size’: 1},],
     # define objectives, and optimization targets
     ’objectives’: [
        {’name’: ’rgb_distance’, ’type’: ’minimum’,}],}],

’bots’: [           # declare available robots
    # choose unique name
    {’name’: ’color_mixer_bob’,
    # declare experimental conditions realizable by the robot
     ’parameters’: [’red’, ’orange’, ’yellow’, ’green’, ’blue’],
    # provide communication information
     ’communication’: {’dump_path’: ’input/’, ’pick_up_path’: ’output/’,}}]}
```

FIG. S.4: ChemOS configuration example for learning the color space. The configuration provides information for all modules of ChemOS, including the learning algorithm, databases, communication interfaces, experimental procedures and automated platforms (bots).

Fig. S.4 presents an example configuration for a color-mixing procedure. Each configuration contains information specific to the individual modules of ChemOS. The color-mixing example in Fig. S.4 selects Phoenics as a learning procedure, and chooses particular settings for this algorithm. Then, the configuration defines the databases for the session, including their paths and backend implementations, which were chosen to be SQLite. The session will be

initialized with two communication channels, slack and Gmail, which are supported simultaneously and can be used at any point during the running session to communicate to ChemOS.

The ‘experiments’ section defines the experimental procedures to be supervised by ChemOS. An experimental procedure is declared with a unique name (‘color-mixing’), experimental conditions (‘variables’) to be modified by ChemOS, and the objectives of the procedure. Note, that an arbitrary number of experimental procedures can be defined in the configuration file, and that ChemOS is able to orchestrate the execution of multiple procedures simultaneously with negligible overhead (see main text for details). Information about available automated platforms is provided in the ‘bots’ section. Again, ChemOS allows for the declaration of multiple automated platforms, and will identify the experimental procedures which can be executed by each of the automated platforms upon initialization of the session. Details for the communication between ChemOS and the automated platforms are defined in the ‘communication’ section. Currently, ChemOS supports communication via local folders, possibly synchronized with dropbox, in addition to the secure copy protocol (SCP) and HTTP communication.

A)

```
# CONFIGURATION FOR LEARNING THE PH SPACE

_SETTINGS = {

    ... identical to color-mixing configuration ...

'experiments': [    # declare experimental procedures
    # choose unique name, define meta-information
    {'name': 'ph-neutralization', 'repetitions': 3, 'max_iter': 25,
     'description': 'Create a solution with pH 7'
     # define experimental conditions for procedure
     'variables':  [
        {'name': 'strong_acid', 'type': 'float', 'low': 0.0, 'high': 50.0, 'size': 1},
        {'name': 'weak_acid',   'type': 'float', 'low': 0.0, 'high': 50.0, 'size': 1},
        {'name': 'neutral',     'type': 'float', 'low': 0.0, 'high': 50.0, 'size': 1},
        {'name': 'weak_base',   'type': 'float', 'low': 0.0, 'high': 50.0, 'size': 1},
        {'name': 'strong_base', 'type': 'float', 'low': 0.0, 'high': 50.0, 'size': 1},],
     # define objectives, and optimization targets
     'objectives': [
        {'name': 'pH_distance', 'type': 'minimum',}],}],

'bots': [           # declare available robots
    # choose unique name
    {'name': 'ph_neutralizer_bob',
    # declare experimental conditions realizable by the robot
     'parameters': ['strong_base', 'weak_base', 'neutral', 'weak_acid', 'strong_acid'],
    # provide communication information
     'communication': {'dump_path': 'input/', 'pick_up_path': 'output/',}}]}
```

B)

```
# CONFIGURATION FOR LEARNING THE DENSITY SPACE

_SETTINGS = {

    ... identical to color-mixing configuration ...

'experiments': [    # declare experimental procedures
    # choose unique name, define meta-information
    {'name': 'densities', 'repetitions': 3, 'max_iter': 25,
     'description': 'Create a solution with a target density of 1 g/mol'
     # define experimental conditions for procedure
     'variables':  [
        {'name': 'density_0.40', 'type': 'float', 'low': 0.0, 'high': 10.0, 'size': 1},
        {'name': 'density_0.55', 'type': 'float', 'low': 0.0, 'high': 10.0, 'size': 1},
        {'name': 'density_0.70', 'type': 'float', 'low': 0.0, 'high': 10.0, 'size': 1},
        {'name': 'density_1.35', 'type': 'float', 'low': 0.0, 'high': 10.0, 'size': 1},
        {'name': 'desnity_1.70', 'type': 'float', 'low': 0.0, 'high': 10.0, 'size': 1},],
     # define objectives, and optimization targets
     'objectives': [
        {'name': 'total_density_difference', 'type': 'minimum',}],}],

'bots': [           # declare available robots
    # choose unique name
    {'name': 'density_equalizer_bob',
    # declare experimental conditions realizable by the robot
     'parameters': ['density_0.40', 'density_0.55', 'density_0.70', 'density_1.35', 'density_1.70'
    # provide communication information
     'communication': {'dump_path': 'input/', 'pick_up_path': 'output/',}}]}
```

FIG. S.5: ChemOS configurations for experimental procedures involving pH measurements (A) and weight measurements (B). Note, that only the ‘experiments’ and the ‘bots’ sections were modified, while all other settings were kept identical to the color-mixing procedure.

The configuration of ChemOS for running a color-mixing experiment only requires little modification to realize the orchestration of a pH or density experiment. Fig. S.5 highlights the required changes to implement these new experiments. While settings regarding the learning procedure or the databases can be identical to the color-mixing configuration presented in Fig. S.4, the ‘experiments’ and ‘bots’ sections need to be adapted to the new procedures.

ChemOS also supports the simultaneous declaration of multiple experimental procedures and/or multiple automated platforms, by simple declarations in the configuration file. Fig. S.6 illustrates these capabilities with an example where two automated platforms, ‘color_mixer_alice’ and ‘color_mixer_bob’ are defined for a color-mixing procedure. Note, that both platforms are capable of realizing the color-mixing procedure, while both platforms can also modify an additional parameter. ChemOS will not trigger these additional parameters when executing a color-mixing experiment. Furthermore, ChemOS automatically implements the communication with ‘color_mixer_alice’ via the secure copy protocol (SCP), while ‘color_mixer_bob’ is expected to be accessible from a local directory, possibly synchronized via dropbox.

The orchestration capabilities of ChemOS to automatically distribute multiple experimental procedures across multiple automated platforms are further illustrated in Fig. S.7. This configuration implements the execution of two different experimental procedures for mixing colors and neutralizing pH, for which three different automated platforms are available, ‘color_mixer_alice’, ‘pH_mixer_bob’, and ‘multi_mixer_charlie’. When a new ChemOS session is started, the experimental parameters defined in the experimental procedures and the robots are analyzed and matched, such that ChemOS identifies which procedures can be executed by with automated platforms. In the example presented in Fig. S.7 ChemOS will automatically determine that the ‘pH-neutralization’ experiment can only be executed by ‘pH_mixer_bob’ and ‘multi_mixer_charlie’, while the ‘color-mixing’ experiment can only be executed on ‘color_mixer_alice’ and ‘multi_mixer_charlie’. After this initial analysis of the declared experimental procedure and automated platforms, ChemOS will only schedule experimental procedures for the qualified automated platforms. This feature of ChemOS disentangles the declaration of experimental procedures and automated platforms. It therefore

```
# CONFIGURATION FOR LEARNING THE COLOR SPACE WITH TWO ROBOTS

_SETTINGS = {

    ... identical to single robot color-mixing configuration ...

'experiments': [    # declare experimental procedures
    # choose unique name, define meta-information
    {'name': 'color-mixing', 'repetitions': 3, 'max_iter': 25,
     'description': 'Create a solution with a target color'
     # define experimental conditions for procedure
     'variables':  [
        {'name': 'red',    'type': 'float', 'low': 0.0, 'high': 1.0, 'size': 1},
        {'name': 'orange', 'type': 'float', 'low': 0.0, 'high': 1.0, 'size': 1},
        {'name': 'yellow', 'type': 'float', 'low': 0.0, 'high': 1.0, 'size': 1},
        {'name': 'green',  'type': 'float', 'low': 0.0, 'high': 1.0, 'size': 1},
        {'name': 'blue',   'type': 'float', 'low': 0.0, 'high': 1.0, 'size': 1},],
     # define objectives, and optimization targets
     'objectives': [
        {'name': 'rgb_distance', 'type': 'minimum',}],}],

'bots': [           # declare available robots
    # choose unique name
    {'name': 'color_mixer_alice',
    # declare experimental conditions realizable by the robot
     'parameters': ['red', 'orange', 'yellow', 'green', 'blue', 'alices_favorite_color'],
    # provide communication information
     'communication': {'username': 'alice', 'host': '192.168.0.1',
                       'dump_path':    '/home/alice/input/',
                       'pick_up_path': '/home/alice/output/',}}

    # choose unique name
    {'name': 'color_mixer_bob',
    # declare experimental conditions realizable by the robot
     'parameters': ['blue', 'orange', 'red', 'green', 'yellow', 'bobs_favorite_color'],
    # provide communication information
     'communication': {'dump_path': 'input_bob/', 'pick_up_path': 'output_bob/',}}]}
```

FIG. S.6: ChemOS configuration for a color-mixing procedure. Note, that two automated platforms are declared, which are both capable of running the color-mixing experiment. While the platform 'color_mixer_bob' is accessible via local directories, possibly synchronized with dropbox, ChemOS communicates with the 'color_mixer_alice' platform via the secure copy protocol (SCP).

simplifies the integration of additional automated platforms into a more complex workflow, and allows the declaration of new experimental procedures without requiring detailed knowledge about the available hardware.

```
# CONFIGURATION FOR RUNNING MULTIPLE EXPERIMENTS ON MULTIPLE ROBOTS

_SETTINGS = {

    ... identical to single robot color-mixing configuration ...

'experiments': [    # declare experimental procedures
    # declare pH neutralization experiment
    {'name': 'pH-neutralization', 'repetitions': 1, 'max_iter': 50,
     'description': 'Create a solution with pH 7',
     'variables':  [
        {'name': 'strong_acid', 'type': 'float', 'low': 0.0, 'high': 25.0, 'size': 1},
        {'name': 'weak_acid',   'type': 'float', 'low': 0.0, 'high': 50.0, 'size': 1},
        {'name': 'weak_base',   'type': 'float', 'low': 0.0, 'high': 50.0, 'size': 1},
        {'name': 'strong_base', 'type': 'float', 'low': 0.0, 'high': 25.0, 'size': 1},],
     'objectives': [
        {'name': 'pH_distance', 'type': 'minimum',}],},

    # declare color-mixing experiment
    {'name': 'color-mixing', 'repetitions': 1, 'max_iter': 50,
     'description': 'Create a solution with a target color',
     'variables':  [
        {'name': 'red',    'type': 'float', 'low': 0.0, 'high': 1.0, 'size': 1},
        {'name': 'orange', 'type': 'float', 'low': 0.0, 'high': 1.0, 'size': 1},
        {'name': 'green',  'type': 'float', 'low': 0.0, 'high': 1.0, 'size': 1},
        {'name': 'blue',   'type': 'float', 'low': 0.0, 'high': 1.0, 'size': 1},],
     'objectives': [
        {'name': 'rgb_distance', 'type': 'minimum',}],}],

'bots': [           # declare available robots
    {'name': 'color_mixer_alice',   # declare color-mixing robot
     'parameters': ['red', 'orange', 'green', 'blue', 'alices_favorite_color'],
     'communication': {'username': 'alice', 'host': '192.168.0.1',
                       'dump_path':    '/home/alice/input/',
                       'pick_up_path': '/home/alice/output/',}}

    {'name': 'pH_mixer_bob',        # declare pH robot
     'parameters': ['strong_base', 'weak_base', 'neutral', 'weak_acid', 'strong_acid'],
     'communication': {'dump_path': 'input_bob/', 'pick_up_path': 'output_bob/',}}

    {'name': 'multi_mixer_charlie', # declare multi-purpose robot
     'parameters': ['red', 'orange', 'green', 'blue',
                    'strong_base', 'weak_base', 'neutral', 'weak_acid', 'strong_acid'],
     'communication': {'dump_path': 'input_charlie/', 'pick_up_path': 'output_charlie/',}}]}
```

FIG. S.7: ChemOS configuration for two experimental procedures and three automated platforms. Based on the unique names of experimental parameters, ChemOS identifies the automated platforms capable of executing experimental procedures upon initialization. In the following, ChemOS will submit experiments only to suitable platforms based on their availability.

S.3. DETAILS ON THE COCKTAIL EXPERIMENTS

We demonstrated the performance of ChemOS with significant interactions between researchers and robots on an experimental procedure for mixing consumable liquids. ChemOS was used to learn recipes of flavorful cocktails by proposing recipes from a learning procedure and receiving feedback on the taste of the cocktail from researchers. Researchers could rate each produced cocktail on a scale from 0 (best) to 4 (worst) in steps of one.

We ran this experimental procedure in two independent sessions with four researchers in each session. Results are depicted in Fig. S.8. We report the ratings of each produced cocktail in panels (A) and (B) for the two different sessions. In both cases, we observe a slight trend of cocktails receiving better (smaller) ratings with more conducted experiments.

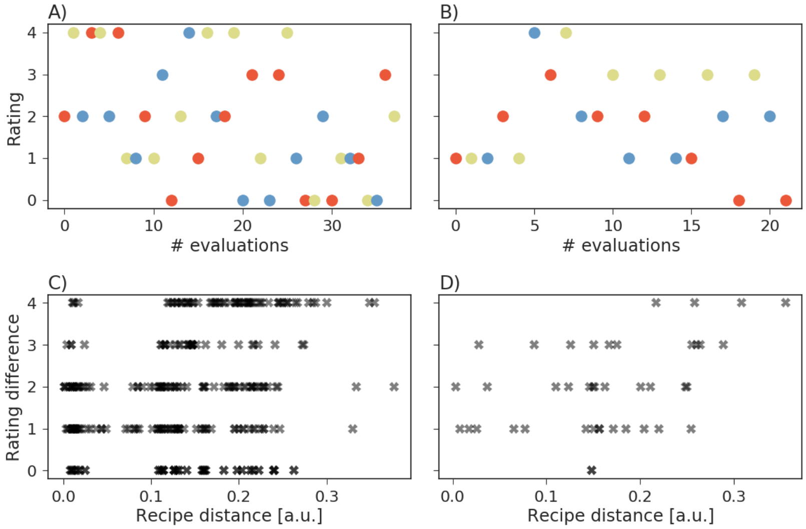

FIG. S.8: Results of the experiments on the Tequila Sunrise space. Panels (A) and (B) present the ratings provided by researchers on produced cocktails for two different sessions. Cocktail recipes were proposed by Phoenics with a bias towards exploration (red), exploitation (blue) or without bias (yellow). Panels (C) and (D) show the distance between recipes with the best rating and all other proposed recipes compared to the difference in the rating for the two separate sessions.

Panels (C) and (D) of Fig. S.8 show the distances in the received ratings between cocktails with the best rating and all other cocktails compared to the distances between the recipes of the cocktails for the two sessions. For both sessions, we observe that the difference in the rating can be large (more than two rating units) even for very small distances in the recipe. This observation indicates that similar recipes can receive rather different ratings. This reflects the subjective nature of the objective function.

S.4. EXTENSIVE CALIBRATION OF A ROBOTIC SEQUENCE FOR DIRECT-INJECT HPLC ANALYSIS

ChemOS is used to run a calibration procedure on a robotics platform for direct-inject HPLC analysis fully autonomously. The goal of the calibration procedure is to find a set of experimental parameters which maximizes the amount of drawn sample reaching the HPLC (see main text for details). We found that the learning procedures implemented in ChemOS quickly find experimental parameters which yield large (> 2500) peak areas. For these experimental procedures, ChemOS was determined to run for a total of 100 iterations.

However, we demonstrate that the workflow established in ChemOS is sufficiently robust to autonomously run a much larger number of experiments. Fig. S.9 displays the results obtained from two calibration runs with 400 and 1000 consecutive experiments each. Depicted are the achieved peak areas with respect to the chosen parameter values for the six parameters P1 to P6 sampled from the random search learning procedure (see main text for details).

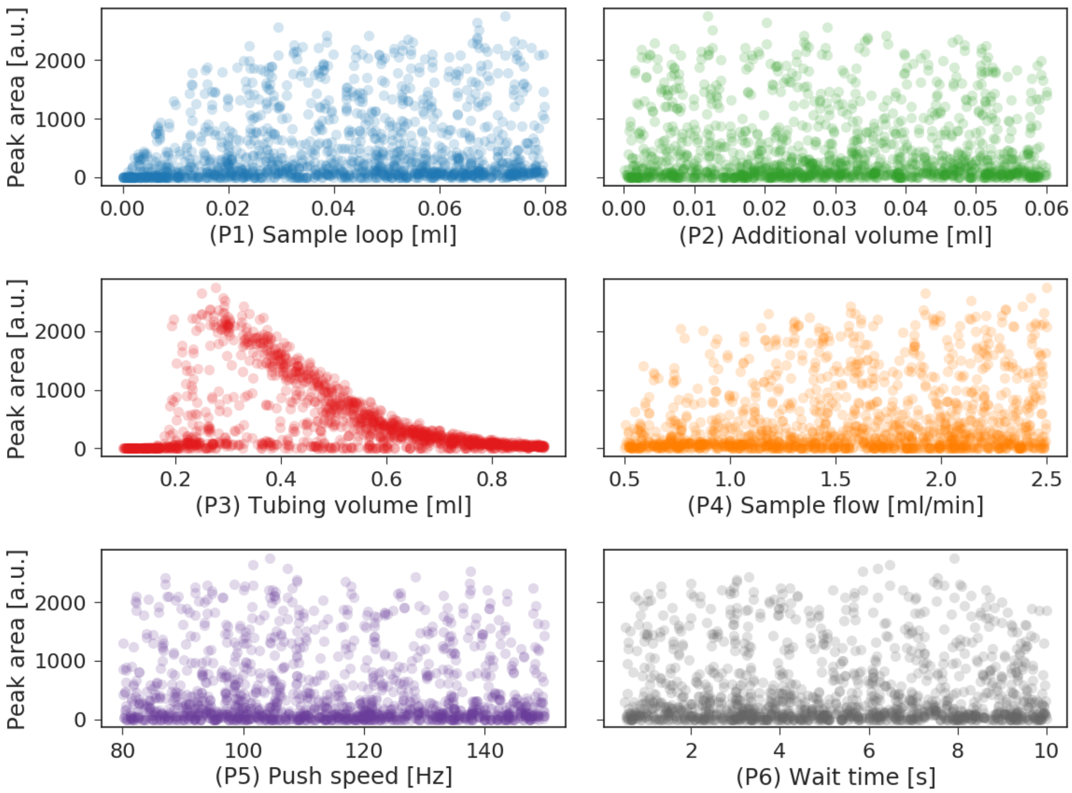

FIG. S.9: Experimental parameters and achieved peak areas obtained from autonomous ChemOS sessions with 400 and 1000 consecutive experiments. We display the peak areas achieved for particular values of individual parameters P1 to P6. Parameter points were sampled from random search.

In the present setup, the magnitude of the response of the detection can be controlled with six distinct tunable parameters such as sample draw, wait time, and the push rates. These parameters are those commonly varied by researchers when optimizing a sampling sequence (see Fig. S.10a). The first parameter (P1) is the volume of the sample loop and internal volume of the 2-way 6-port valve. The second parameter (P2) is the volume required to draw the sample to the 2-way 6-port valve. This volume is commonly combined with the first volume in most sampling sequences, and they are separated here to allow for additional sample manipulation outside of the sampling sequence. The third parameter (P3) is the volume required to drive the sample plug from the sample loop, through the in-line mixer, and to the second valve. The fourth and fifth parameters (P4, P5) control the draw rate and the push rate of the sample and push pumps respectively. The sixth parameter (P6) is the time waited after drawing sample and before switching the first selection valve. This allows time for equilibration of any cavitation bubbles in the sample line and syringe.

We observe that the achieved area is mostly influenced by parameter P3, which controls the amount of volume required to push the sample plug from the sample loop to the second valve. Parameters P1 and P4 mildly influence the achieved peak areas, with larger parameter values resulting in larger peak areas. The other three parameters show no significant influence on the peak area.

[1] F. Häse, L. M. Roch, C. Kreisbeck, and A. Aspuru-Guzik. Phoenics: A Bayesian optimizer for chemistry. *ACS Centr. Sci.*, 4:1134–1145, 2018.

[2] F. Hutter, H. Hoos, and K. Leyton-Brown. Sequential Model-Based Optimization for General Algorithmic Configuration. In *International Conference on Learning and Intelligent Optimization*, volume 5, 2011.

[3] F. Hutter, H. Hoos, and K. Leyton-Brown. Learning and Intelligent Optimization. In *Learning and Intelligent Optimization*, page 55, 2012.

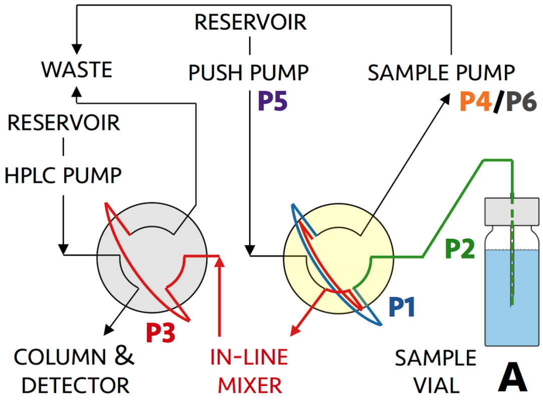

FIG. S.10: Sketch of the automated robotic sequence for direct-inject HPLC sampling. The setup is controlled by six parameters (P1-P6), which are explained in detail in the main text.

[4] M. Lindauer, K. Eggensperger, M. Feurer, S. Falkner, A. Biedenkapp, and F. Hutter. Smac v3: Algorithm configuration in python. `https://github.com/automl/SMAC3`, 2017.

[5] J. Snoek, H. Larochell, and R. P. Adams. Practical Bayesian optimization of machine learning algorithms. In *Advances in Neural Information Processing Systems (NIPS)*, volume 25, pages 2951–2959. 2012.

[6] J. Snoek, K. Swersky, R. Zemel, and R. P. Adams. Input warping for bayesian optimization of non-stationary functions. In *International Conference on Machine Learning*, pages 1674–1682, 2014.

[7] J. A. Bergstra, R. Badenet, Y. Bengio, and B. Kégl. Algorithms for hyper-parameter optimization. In *Advances in Neural Information Processing Systems (NIPS)*, volume 24, pages 2546–2554, 2011.

[8] J. Bergstra and Y. Bengio. Random Search for Hyper-Parameter Optimization. *J. Mach. Learn. Res.*, 13:281–305, 2012.

[9] L. Breiman. Random forests. *Machine learning*, 45:5, 2001.

[10] R. Martinez-Cantin, N. de Freitas, E. Brochu, J. Castellanor, and A. Doucet. A Bayesian exporation-exploitation approach for optimal online sensing and planning with a visually guided mobile robot. *Auton. Robots*, 27:93, 2009.

[11] M. A. Osborne, R. Garnett, and S. J. Roberts. Gaussian processes for global optimization. In *3rd international conference on learning and intelligent optimization (LION3)*, pages 1–15, 2009.

[12] T. Desautels, A. Krause, and J. W. Burdick. Parallelizing exploration-exploitation tradeoffs in Gaussian process bandit optimization. *J. Mach. Learn. Res.*, 15(1):3873–3923, 2014.

[13] Steven Bird, Ewan Klein, and Edward Loper. *Natural language processing with Python: analyzing text with the natural language toolkit.* "O'Reilly Media, Inc.", 2009.

[14] Martín Abadi, Ashish Agarwal, Paul Barham, Eugene Brevdo, Zhifeng Chen, Craig Citro, Greg S. Corrado, Andy Davis, Jeffrey Dean, Matthieu Devin, Sanjay Ghemawat, Ian Goodfellow, Andrew Harp, Geoffrey Irving, Michael Isard, Yangqing Jia, Rafal Jozefowicz, Lukasz Kaiser, Manjunath Kudlur, Josh Levenberg, Dan Mané, Rajat Monga, Sherry Moore, Derek Murray, Chris Olah, Mike Schuster, Jonathon Shlens, Benoit Steiner, Ilya Sutskever, Kunal Talwar, Paul Tucker, Vincent Vanhoucke, Vijay Vasudevan, Fernanda Viégas, Oriol Vinyals, Pete Warden, Martin Wattenberg, Martin Wicke, Yuan Yu, and Xiaoqiang Zheng. TensorFlow: Large-scale machine learning on heterogeneous systems, 2015. Software available from tensorflow.org.

[15] Yuan Tang. TF. Learn: TensorFlows High-level Module for Distributed Machine Learning. *arXiv preprint arXiv:1612.04251*, 2016.

[16] Diederik P Kingma and Jimmy Ba. Adam: A method for stochastic optimization. *arXiv preprint arXiv:1412.6980*, 2014.
